# Genome-Wide Analysis of the Peroxidase Gene Family and Verification of Lignin Synthesis-Related Genes in Watermelon

**DOI:** 10.3390/ijms23020642

**Published:** 2022-01-07

**Authors:** Tiantian Yang, Pengyu Zhang, Jiahui Pan, Sikandar Amanullah, Feishi Luan, Wenhao Han, Hongyu Liu, Xuezheng Wang

**Affiliations:** 1 College of Horticulture and Landscape Architecture, Northeast Agricultural University, No. 600, Changjiang Road, Harbin 150030, China; yttneau2021@gmail.com (T.Y.); zpy1043804675@gmail.com (P.Z.); jhpneau2021@gmail.com (J.P.); sikandaraman@yahoo.com (S.A.); luanfeishi@neau.edu.cn (F.L.); hwh@neau.edu.cn (W.H.); 2 Key Laboratory of Biology and Genetic Improvement of Horticulture Crops (Northeast Region), Ministry of Agriculture and Rural Affairs, Harbin 150030, China

**Keywords:** watermelon, peroxidase, genome-wide identification, transcriptome analysis, peel hardness, qRT-PCR

## Abstract

Watermelon (*Citrullus lanatus*) is an important horticultural crop worldwide, but peel cracking caused by peel hardness severely decreases its quality. Lignification is one of the important functions of class III peroxidase (PRX), and its accumulation in the plant cell wall leads to cell thickening and wood hardening. For in-depth physiological and genetical understanding, we studied the relationship between peel hardness and lignin accumulation and the role of PRXs affecting peel lignin biosynthesis using genome-wide bioinformatics analysis. The obtained results showed that lignin accumulation gradually increased to form the peel stone cell structure, and tissue lignification led to peel hardness. A total of 79 ClPRXs (class III) were identified using bioinformatics analysis, which were widely distributed on 11 chromosomes. The constructed phylogenetics indicated that ClPRXs were divided into seven groups and eleven subclasses, and gene members of each group had highly conserved intron structures. Repeated pattern analysis showed that deletion and replication events occurred during the process of ClPRX amplification. However, in the whole-protein sequence alignment analysis, high homology was not observed, although all contained four conserved functional sites. Repeated pattern analysis showed that deletion and replication events occurred during ClPRXs’ amplification process. The prediction of the promoter cis-acting element and qRT-PCR analysis in four tissues (leaf, petiole, stem, and peel) showed different expression patterns for tissue specificity, abiotic stress, and hormone response by providing a genetic basis of the ClPRX gene family involved in a variety of physiological processes in plants. To our knowledge, we for the first time report the key roles of two ClPRXs in watermelon peel lignin synthesis. In conclusion, the extensive data collected in this study can be used for additional functional analysis of ClPRXs in watermelon growth and development and hormone and abiotic stress response.

## 1. Introduction

The class III peroxidase gene family (PRXs) is a widely distributed isozyme family type that is well known to have various short names, e.g., PRX, POD, POX, and PER, and contributes to multiple significant physiological reactions in plants [1]. Its key functions are involved in causing reduction–oxidation reactions in electrons triggered by H_2_O_2_ and other types of organic and inorganic compounds, e.g., eliminating the excess amount of H_2_O_2_ produced in plant tissue, facilitating wound healing, cross-linking small molecules of oxidized poly-lignin within the cell wall, and providing protection against destructive insects and pathogens [2,3,4].

Multiple studies have revealed that different types of plants contain a large number of PRXs with different contributions, e.g., 73 PRX genes in Arabidopsis [5], 138 PRX genes in rice [6], 119 PRX genes in maize [7], 94 PRX genes in pear [8], 91 POD genes in cassava [9], 102 PRX genes in potato [1], and 90 POD genes in birch [10], among others. The proteins formed by these genes exhibit highly conserved amino acid motifs that include two conserved histidine motifs with chemical binding sites for heme, distal histidine vital for catalytic activity, and eight cysteines that interact to form constant disulfide bonds and are essential amino acids in the secondary structure of peroxidases [11]. However, the amino acid sequences of all these genes are highly conserved, and the protein structure and molecular weight are similar between the lineal and adjacent homologous genes; however, the presence of multiple catalytic forms of peroxidases suggests that these peroxidases may be functional in specialized forms [12]. Therefore, genome-wide analysis of these polygene families would be very helpful to better understand their gene composition and the characteristics of the protein structure, as well as to explain the relationship between differential peroxidase genes and physiological characteristics.

One of the typical functions of PRXs is the accumulation of lignin in the cell wall, which significantly triggers cell wall thickening and hardening [13] and was found to be expressed in multiple tissues of roots, stems, leaves, and fruits. Plant-specific peroxidase (EC1.11.1.7) is considered the final reaction enzyme catalyzed by the polymerization of lignin into macromolecules (coumaryl alcohol, coniferyl alcohol, and sinapyl alcohol), and this lignification process occurs throughout the growth of plants [14,15]. In Arabidopsis, the *AtPrx33* and *AtPrx34* genes are associated with root elongation [12]. *AtPrx2*, *AtPrx25*, *AtPrx71*, and *AtPrx72* play the main role in lignification in Arabidopsis stems and stabilize the plant vasculature in addition to serving as barriers against microbial infections [16,17]. The expression of an antisense cationic peroxidase gene (*Shpx6a*) construct was effective in reducing peroxidase activity in transgenic poplar leaves [18]. The *GhPOX1* gene is involved in fiber elongation in cotton by maintaining high levels of reactive oxygen species [19]. The *PbPRX2* gene has a key role in lignin polymerization in Chinese pear fruit [20]. Watermelon peel is the most important protective tissue of fruit, and fruit with a high peel hardness is often characterized by difficulty cracking, a long storage time, and safe transport. In a previous study conducted in our laboratory, it was found that the peel of crack-susceptible watermelon cultivar “812” contains no stone cell structure, low lignin accumulation, and a low peel hardness of ripe fruit as compared to the crack-resistant watermelon cultivar “1061” [21]. Therefore, we speculated that peroxidase genes might be involved in the lignin synthesis process of watermelon peel.

At present, molecular and genetic studies are lacking on ClPRXs involved in peel lignin accumulation in watermelon peel. Therefore, the core objective of the present study was to carry out a systematic and comprehensive bioinformatics analysis of ClPRXs by preliminary screening of PRX genes, mapping of structural and chromosomal regions, comparative sequence homology, assessment of gene duplication evolutionary history, cis regulatory element analysis, prediction of the functional domain of conserved proteins and subcellular localization, functional enrichment analysis, and promoter element analysis, together with the analysis of two contrasting watermelon materials with obvious differences in peel hardness, peel lignin accumulation, and peel lignin quantification at different periods to predict the genes involved in watermelon lignin synthesis. We further performed physiological analysis, fruit transcriptome expression level analysis, and gene expression verification during different developmental periods using real-time quantitative PCR (qRT-PCR). This study will facilitate new insights for the further study of the functional mechanism of ClPRXs.

## 2. Results

### 2.1. Identification of ClPRX Gene Family Members

In this study, 74 candidate peroxidase genes were obtained by keyword search, 79 by HMMER search, and 376 by BLASTP search (65 after weight removal). The 79 candidate peroxidase genes were finally screened by further deduplication, motif analysis, and functional domain analysis. The inferred genes obtained by HMMER (PF00141) were characterized by conserved and stable structures, and the searched genes contained two other kinds of results. The *Cla97C01G007780* gene was obtained by keyword search and removed due to the absence of multiple motifs and the absence of a conserved peroxidase domain.

The 79 peroxidase genes were named *ClPRX01* to *ClPRX79* based on their genetic location across the watermelon chromosome. All these genes were distributed alone or in clusters on eleven chromosomes, among which chromosomes 4 and 10 had the least distribution with only two counts. Chromosome 2 showed the most abundant genes, with a maximum number of 22 counts. The length of the coding region was 966–14,306 bp, the length of the CDS region was 753–7195 bp; the number of exons was 1–14; the coding protein size was 250–632 aa; the molecular weight was 27,274.98–71,578.44 Da; the PI value was 4.42–9.57 (there were 36 acidic proteins of PI < 7 and 43 basic proteins of PI > 7). The secondary structure was mainly characterized as an irregular wavy pattern, accounting for 46.84–63.60%. The specific information for the corresponding numbering, chromosome location, gene starting and ending locations, coding region length, CDS length, number of exons, and encoded protein characteristics of each gene is shown below in Table 1.

### 2.2. Chromosomal Localization and Gene Replication Relationship of ClPRXs

**Figure 1:**
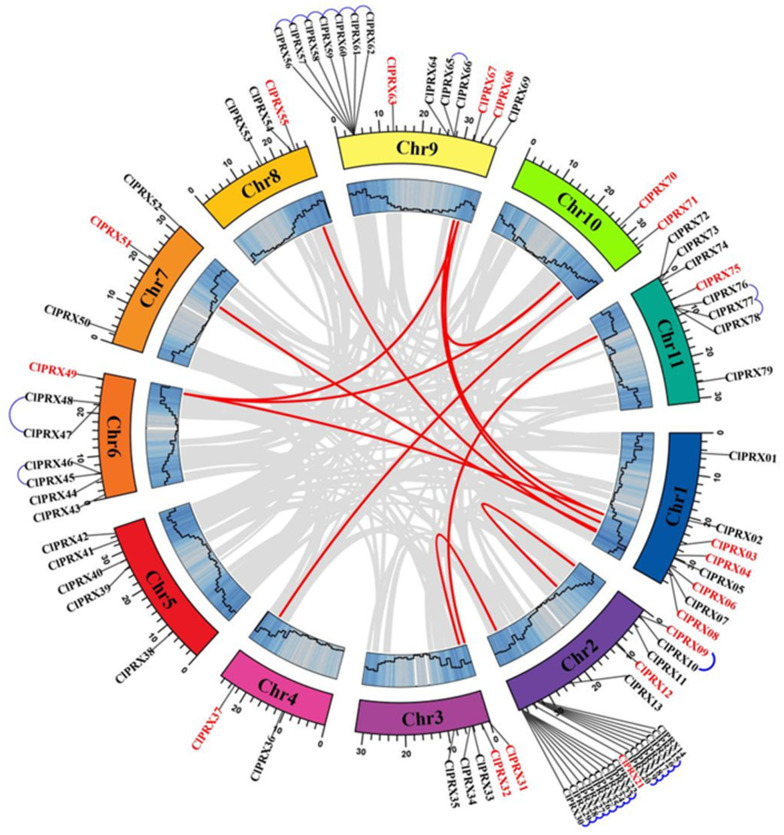
A Circos diagram of ClPRXs’ localization on each chromosome and their linear relationship between genes. Fragment replication of gene pairs is linked by red lines and tandem replication of gene pairs by blue lines.

A total of 79 ClPRXs were widely distributed over 11 chromosomes (Figure 1). Two chromosomes (4 and 10) exhibited the least distribution with the presence of two gene counts, and chromosomes 7 and 8 showed three gene counts. There was a total of five genes on chromosomes 3 and 5, and chromosome 6 showed the presence of seven genes. There was a maximum of eight genes on chromosome 11, and chromosome 9 had fourteen genes; however, chromosome 2 had the widest distribution, with 22 genes. Among them, genes on chromosomes 1, 2, 6, 9, and 11 were distributed in cluster form, with relatively dense genes.

The internal linearity of the97,103 watermelon genome species showed that ClPRXs displayed 12 pairs of fragment duplicated genes (Table S2) and 25 pairs of tandem duplicated genes (Table S3). After the calculation of the non-synonymous mutation rate (Ka) and synonymous mutation rate (Ks) values, two pairs of fragment duplicated genes (*ClPRX03-ClPRX49* and *ClPRX04-ClPRX67*) were shown whose Ks value was zero All the gene pairs with synonymous mutations also had Ka/Ks ratios of less than one. The results indicated that the intraspecific evolution of ClPRXs existed by purified selection, which was relatively conservative in evolution, eliminating harmful mutations and keeping the translated proteins of ClPRXs unchanged.

### 2.3. Interspecific Collinearity of PRXs

**Figure 2:**
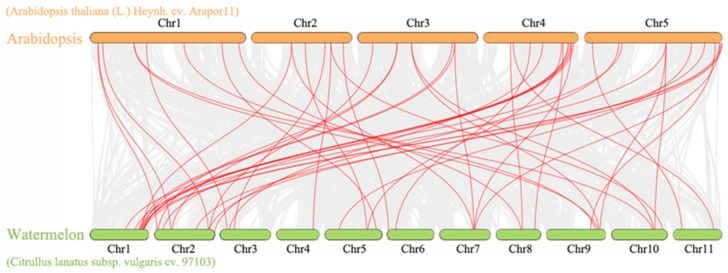
Comparative linear relationship of PRXs in watermelon and Arabidopsis.

The collinearity analysis results between watermelon and Arabidopsis revealed 13,489 collinearity gene pairs between the two species (Figure 2), among which 35 PRX genes of Arabidopsis and 28 ClPRXs constituted 47 collinearity gene pairs (Table S4). The colinear gene pairs of ClPRXs reached a maximum (11 pairs) on Chr01. The number of gene pairs on Chr02, Chr09, Chr10, Chr05, Chr07, Chr03, Chr08, Chr06, Chr011, and Chr04 decreased sequentially, but Chr04 had at least one gene pair. These genes showed high similarity with the same function and might originate from a common ancestor. In addition, we found that Arabidopsis PRX genes can map 1–4 ClPRX homologous genes, indicating that ClPRXs may have fourfold replication in the evolutionary process [22]. After the Ka and Ks value calculations, thirteen pairs of collinear peroxidase genes of watermelon and Arabidopsis with a Ks value of zero were found. Synonymous mutations had occurred and could be synonymous mutations. The remaining genes for Ka/Ks had a value less than one, which showed more conservative results for the ClPRXs in the interspecific evolution method for the purification of choice. The gene pairs with a stable structure and collinearity between species were also more consistent in function.

### 2.4. ClPRXs’ Evolutionary Tree, Gene Structure, Protein Sequence Comparison, and Conserved Structure

**Figure 3:**
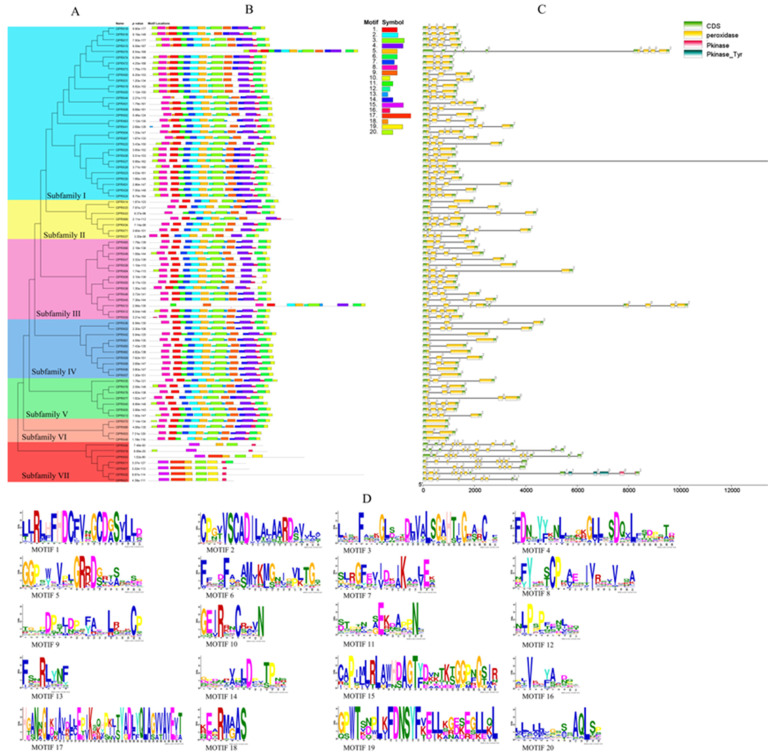
Constructed phylogenetic tree, gene structure, and conserved protein structure of ClPRXs. (**A**) Intraspecific evolutionary tree of ClPRX members. (**B**) The distribution of conserved motifs of ClPRX proteins. (**C**) The intron–exon structure and functional domain of the conserved structure of ClPRXs. (**D**) The conserved motifs of ClPRX proteins.

The constructed phylogenetic tree of ClPRXs showed division into seven groups (Figure 3A). The number of genes in group I was the largest (30 counts), followed by group III (14 counts), group IV (10 counts), groups II, V, and VII (7 counts), group VI (4 counts), and finally, group IV (4 counts). The gene structure, amino acid ratio, conserved motif elements, and functional domain similarity of the genes were higher in the group. The gene exon–intron structure is an important feature of gene evolution, but the exon structure is a significant part of the coding protein. The insertion of introns or structural loss in the process of exon evolution might lead to positive selection, functional loss, or mutation in gene evolution. There were significant differences in the number of exons in ClPRXs, as shown in Figure 3C. The number of exons in 79 ClPRXs ranged from one to fourteen, among which sixty-nine genes had three and four exons and were the most common genes. The structure of the four genes in group VI was relatively stable, with only one to two exons, and three of them had no intrusions. The seven genes in group VII showed the most complex genetic structure, with a maximum of fourteen exon counts in *ClPRX32* gene members and the fewest exons with nine counts in *ClPRX21*.

**Figure 4:**
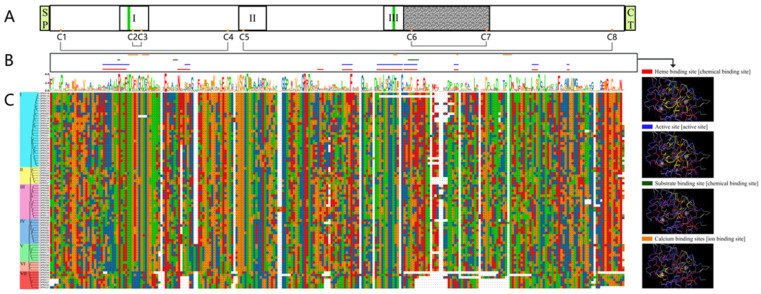
Structural characteristics of ClPRX protein. (**A**) Schematic diagram of the primary structure of the class III peroxidase. The green boxes at the front and back signify the N-terminal signal peptide (SP) and c-terminal extension domain (CT). The middle three boxes represent the highly conserved domain: box I is the distal-heme-binding site domain; box Ⅱ is the unknown functional domain; box Ⅲ is the proximal-heme-binding site domain. The gray area with obvious variation was assumed to be a variable domain responsible for the specific catalytic function of peroxidase. The highly conserved distal histidine (Hd) and proximal histidine (HP) are heme-binding sites represented by green bars. Eight cysteines (C1–C8) are key amino acids that form disulfide bonds to form the secondary structure of peroxidase, which are represented by yellow solid circles. The four conserved disulfide bonds formed are shown as black lines in plane. (**B**) represents the four conserved domains and site location information of the third type of peroxidase, which are marked on the amino acid sequence with four different colors, and the 3D model of the structure of the four domains is displayed, the yellow part being the functional site. (**C**) is the comparison results of 7 groups of ClPRX protein sequences.

To display the clear protein structural characteristics of ClPRXs, we combined amino acid comparative, protein structural characteristic, and protein functional domain binding site analyses. In accordance with previous studies, we found that ClPRXs had three highly conserved protein domains (I, II, and II, as shown in Figure 4A), which were the distal-heme-binding domain, position domain, and proximal-heme-binding domain, and these domains also corresponded to the conserved protein motifs Motif 1, Motif 2, and Motif 3 in Figure 3D, respectively. From the comparison of the protein sequences of ClPRXs in Figure 4C, the region after the conserved domain of III demonstrated a large difference due to the peroxidase characteristics in the multifunction catalysis. This region might be the catalytic domain specific to the peroxidase (shown as the gray area in Figure 4A). Peroxidase has four main conserved functions, and each functional site is marked in different colors, as shown in Figure 4B. The heme-binding site mainly consists of distal histidine (Hd) and proximal histidine (HP), and the proximal histidine (HP) can connect one Fe element. In addition, the active site, substrate-binding site, and Ca-binding site are responsible for connecting two calcium elements.

### 2.5. Comparative Analysis of PRXs between Watermelon and Arabidopsis

**Figure 5:**
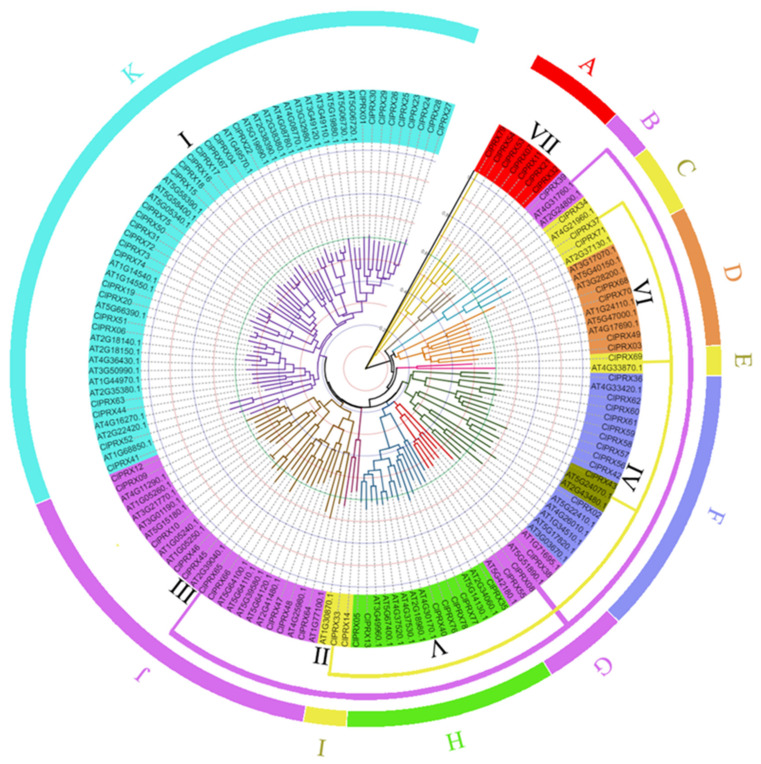
Phylogenetic tree analysis of PRXs in watermelon and Arabidopsis.

The phylogenetic tree of all genes was constructed and analyzed between the watermelon and Arabidopsis PRX families (Figure 5), and the tree results were mainly divided into seven large groups and eleven subclasses. According to the results of the evolutionary tree analysis, the PRX gene families of watermelon and Arabidopsis could be classified into 11 subclasses (A, B, C, D, E, F, G, H, I, J, K) to better display the evolutionary and functional relationships between watermelon and Arabidopsis PRX genes. According to the internal evolutionary relationship of ClPRXs, we divided them into seven groups (Figure 3A). When constructing the evolutionary tree with the Arabidopsis PRX gene family, we found that genes of group II and III ClPRX members had evolutionary relationship changes and showed a scattered distribution. Group II was divided into three subclasses (C, E, and I), and group III was divided into three subclasses (B, G, and J). All seven groups are indicated in Figure 5.

The ClPRXs showed a unique group VII (also subclass A) in watermelon, and group VII had the most distant evolutionary relationship. In accordance with the results shown in Figure 3A, compared with the other six groups, the gene structure of this group contained a peroxidase conserved domain; however, the number of introns in their gene structure was very large, and their conserved protein motifs were mostly missing. There were multiple pairs of homologous genes between PRXs of watermelon and Arabidopsis. The maximum PRXs of subclass K were 30 ClPRX genes and 27 AtPRX genes. Subclass E, subclass I, and subclass B were isolated from group II. Group III contained fewer genes, among which subclass E contained the minimum number of PRX genes, including one ClPRX gene and one AtPRX gene. However, subclass I and subclass B contained three PRX genes, and subclass I contained two ClPRX genes and one AtPRX gene. Subclass B included one ClPRX gene and two AtPRX genes. As shown in Figure 5, the phylogenetic tree was subdivided into 11 subclasses of watermelon and Arabidopsis PRX gene families. The same subclass showed the closest evolutionary relationship and had the most similar functions. The functions of unknown ClPRX members could be further predicted and verified based on the published functions of Arabidopsis PRX gene family members.

### 2.6. Prediction of Subcellular Localization of ClPRX Proteins

As shown in the attached Table S5, ClPRX proteins were mainly located in the chloroplast and cytoplasm. The first six groups of proteins were mainly localized in chloroplasts, which exceeded 82% of the full predicted value of ten. The subcellular localization results for the seventh group of proteins were different and were obviously divided into three types: *ClPRX54*, *ClPRX79*, and *ClPRX53*. These proteins were mainly located in chloroplasts with a predictive integral value of 5.87, followed by cytoplasm with a predictive integral value of 2.67. The subcellular localization of the *ClPRX11*, *ClPRX07*, and *ClPRX21* proteins was mainly in the cytoplasm with predicted integral values of 4.31, 4.25, and 5.03, respectively, and the *ClPRX11*, *ClPRX07*, and *ClPRX21* proteins were in vacuoles with predicted integral values of 2.81, 2.97, and 2.85, respectively. The subcellular localization of the *ClPRX32* protein was mainly in the cytoplasm and nucleus with predicted integral values of 4.22 and 4.15, respectively.

### 2.7. Functional Gene Ontology and Kyoto Encyclopedia of Genes and Genomes Enrichment of ClPRXs

**Figure 6:**
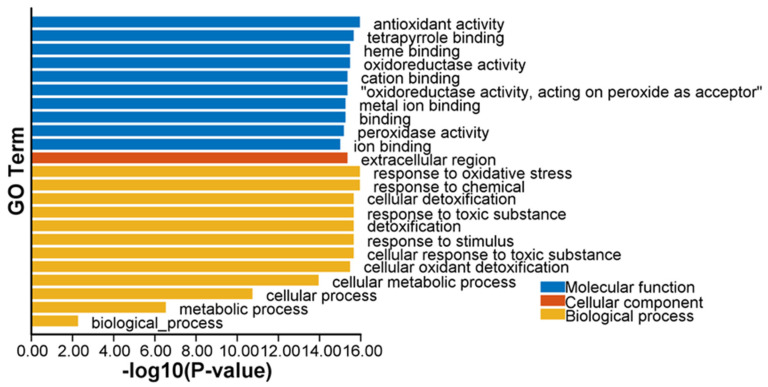
GO enrichment analysis of ClPRXs in watermelon.

Gene Ontology (GO) enrichment analysis of all ClPRXs (Figure 6) showed that the cellular component was mainly the extracellular region, accounting for 71 out of 79. *ClPRX07*, *ClPRX10*, *ClPRX11*, *ClPRX21*, *ClPRX32*, *ClPRX53*, *ClPRX54*, and *ClPRX79* were not enriched. All the PRX genes of watermelon were enriched in the GO function of binding in the biological process. The main enriched functions were functional antioxidant activity, tetrapyrrole binding, heme binding, oxidoreductase activity, and oxidoreductase activity, with peroxide acceptor activity accounting for 78 out of 79 total functions, and neither *ClPRX32* was enriched. The next most enriched functions were ion binding (total of 73 out of 79), cation binding, and metal ion binding (total of 72 out of 79). Among the molecular functions, all the PRX genes of watermelon were enriched in response to stimulus, cellular process, and biological process. Seven functions were prominently enriched: response to oxidative stress, response to chemicals, cellular detoxification, response to toxic substances, detoxification, cellular response to toxic substances. Cellular oxidant detoxification accounted for 78 out of 79 functions, while *ClPRX32* was not detected at all. Cellular metabolic processes and metabolic processes accounted for 74 out of 79 functions. The specific PRX gene GO enrichment information is provided in Table S6.

**Figure 7:**
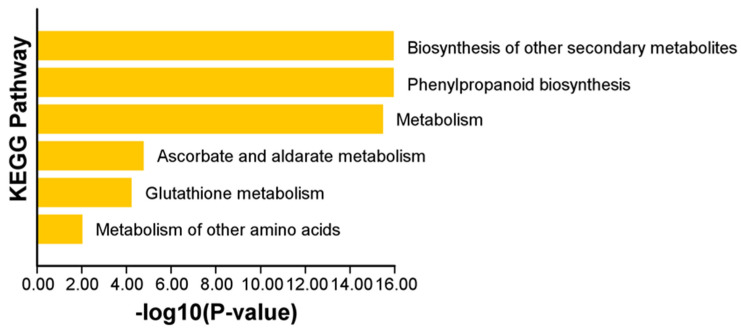
KEGG enrichment analysis of ClPRXs in watermelon.

Kyoto Encyclopedia of Genes and Genomes (KEGG) enrichment analysis showed that only seventy-five genes out of the seventy-nine ClPRXs were hits, and the main class was involved in six metabolic pathways (Figure 7): the biosynthesis of other secondary metabolites. The phenylpropanoid biosynthesis pathway accounted for 68 out of 75 metabolites: seventy-four metabolites participated in the metabolic pathway, and six out of seventy-five were mainly involved in metabolism, ascorbate and aldarate metabolism, glutathione metabolism, and the metabolism of other amino acids. The subsequent main classes, A09130 Environmental Information Processing, A09150 Organismal Systems, A09180 Brite Hierarchies, A09130 Environmental Information Processing, and A09180 Brite Hierarchies were removed because of a *p*-value > 0.05. According to the KEGG enrichment analysis of seventy-nine ClPRXs, four (*ClPRX16*, *ClPRX42*, *ClPRX56*, and *ClPRX61*) were not involved in any pathway. The KEGG enrichment information for the specific PRX gene families is shown in the attached Table S7.

### 2.8. Promoter Elements of ClPRXs

**Figure 8:**
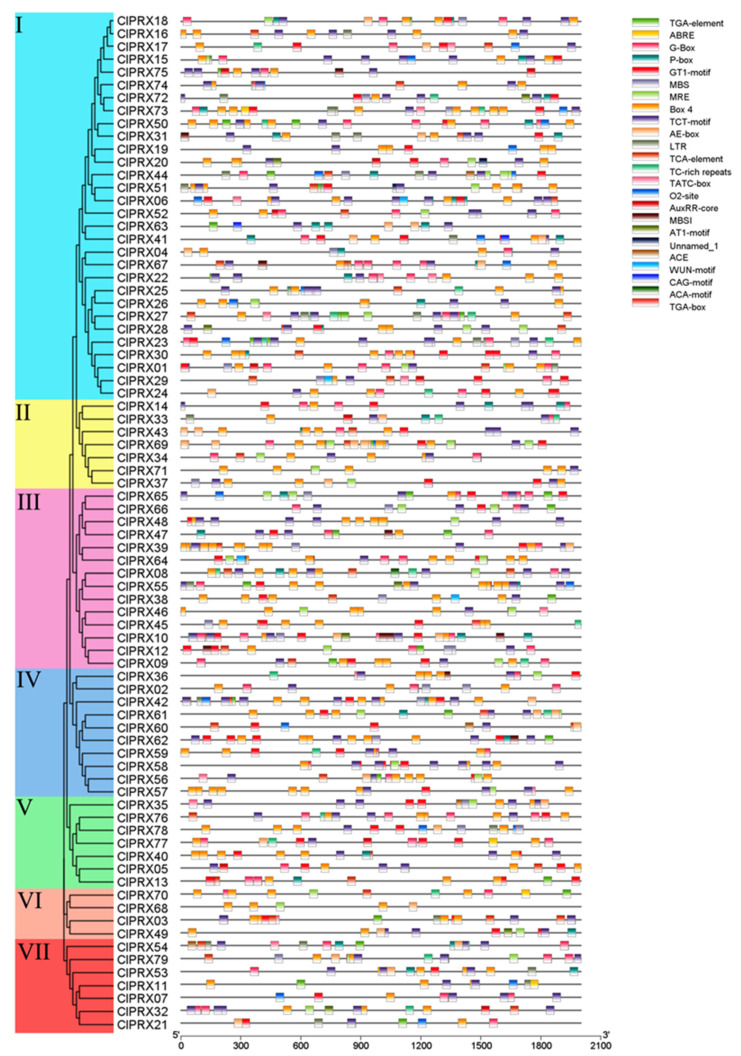
The cis-acting element analysis of the ClPRXs’ promoter.

According to the promoter analysis of ClPRX convenience elements (Figure 8), the family genes were mainly involved in environment response (photosensitive, low temperature, drought), hormone (auxin, gibberellin, abscisic acid, salicylic acid) regulation, growth and development (zein metabolic regulation, the biosynthesis of flavonoids), and plant defense and stress reactions, among other activities. It is involved in anti-stress, light response elements (ACE, Unnamed_1, G-box, GT1-Motif, MRE, Box 4, CAG-motif, TCT-motif, AT1-motif, AE-box, and ACA-motif were involved in 5, 2, 53, 60, 34, 77, 2, 73, 7, 25, and 1 genes, respectively). Twenty-two genes were involved in low-temperature response elements (LTRs).

From Table S2, 28 genes were involved in the auxin core, TGA box, and TGA element, which are involved in hormone regulation. Fourteen and thirty-three genes were involved in gibberellin-responsive elements (TATC-box and P-box), respectively. There were 40 genes involved in salicylic acid response elements (TCA elements). There were 48 genes involved in ABRE and 23 genes involved in cis-acting regulatory elements participating in zein metabolism regulation (O2-site). Eight genes were involved in MYB binding sites participating in flavonoid biosynthetic gene regulation (MBSI). The cis-acting element was involved in defense and stress responsiveness (TC-rich repeats) and water responsiveness. Element WUN-motif was made up of twenty-nine and four genes, respectively. Information on the promoter homeopathic elements of specific genes is shown in Table S8.

### 2.9. Expression Analysis of ClPRXs in Watermelon Fruits

**Figure 9:**
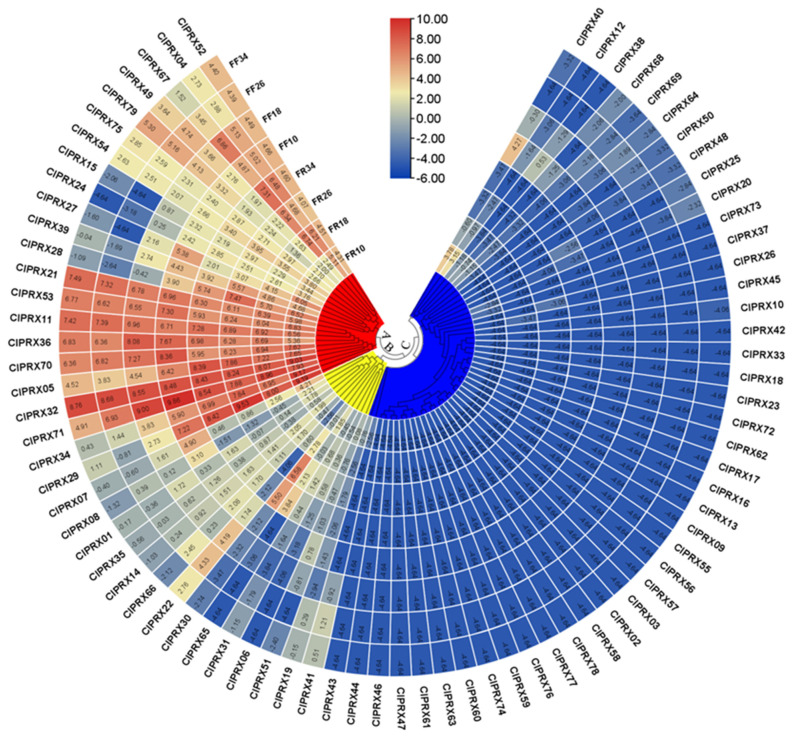
Expression analysis of *ClPRXs* in watermelon peel and flesh at different stages.

Transcriptome data of watermelon fruits at different periods were used to analyze the expression levels of ClPRXs in fruit peel and flesh, and the results showed that 51 genes were expressed in fruit tissues (Figure 9). Gene heatmaps were drawn according to the expression levels, and cluster analysis of PRX gene expression was performed by the expression patterns of ClPRX fruits, which were divided into three large groups (A, B, and C). Group A exhibited highly expressed genes, containing 22 PRX genes, and Group B contained genes with low expression, including 12 PRX genes. Group C contained 45 genes, including genes with no and weak expression. Peroxidase (EC1. 11. 1.7) is considered to be the last step of polymerization in the lignin macromolecule synthesis enzyme reaction because the lignification process can effectively enhance skin hardness, reducing dehiscent fruit, and our results showed that groups A and B contained 34 genes that might be involved in lignin accumulation and some key genes involved in the development of watermelon fruit, which mainly affected the formation of the watermelon peel stone cell structure [14,15].

### 2.10. Observation of the Microstructure, Hardness, and Lignin Content of Watermelon Peel at Different Periods

**Figure 10:**
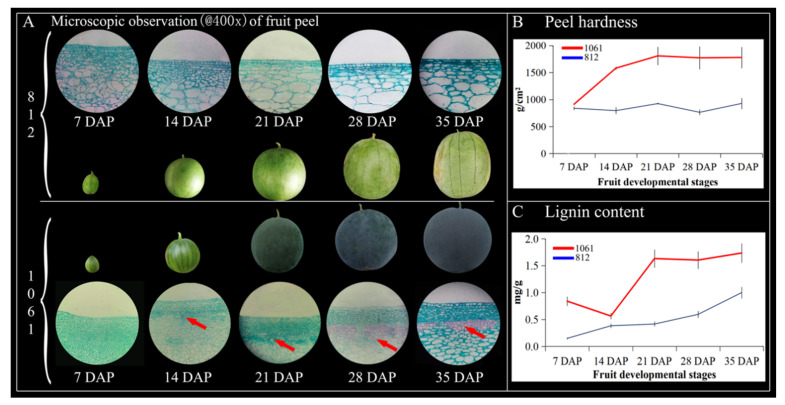
Watermelon peel microstructure (**A**); red bold arrows indicate the formation of the stone cell structure. Peel hardness of watermelon materials (**B**); and lignin contents of watermelon peel materials (**C**); red and blue lines represent the differences in the values of the means ± SD bars, and bold red arrows indicated the stone cell structure formation in the 1061 and 812 materials, respectively.

There were significant differences in peel structure development, peel hardness, and lignin content accumulation in crack resistance at different developmental stages of two watermelon materials. There was also a noteworthy correlation among peel hardness, structure, and endogenous lignin contents (Figure 10). The peel microstructure analysis of crack-susceptible watermelon “812” displayed no presence of the stone cell structure, while the peel of material “1061” was crack-resistant because there was the presence of a cell structure, which began to develop at 14 DAP (Figure 10A). We also noticed that peel hardness (g/cm^2^) of crack-susceptible watermelon “812” did not change relatively (838.87 ± 31.38, 794.43 ± 61.37, 924.80 ± 11.05, 763.57 ± 48.21, 927.17 ± 98.70), but the peel hardness of crack-resistant material “1061” (912.83 ± 10.41, 1580.47 ± 27.14, 1807.73 ± 163.51, 1771.67 ± 206.01, 1778.17 ± 192.75) changed steadily due to the development of the cellular structure and lignification accumulation, which exhibited a clear correlation with peel hardness at three key periods of 14 DAP, 21 DAP, and 28 DAP (Figure 10B), but hardness seemed to be stable until 35 DAP.

Further, we measured the endogenous lignin contents (mg/g) of both watermelon materials (Figure 10C) and observed that the lignin content of the fruit peel of crack-resistant material “1061” was significantly higher (0.84 ± 0.07, 0.56 ± 0.06, 1.63 ± 0.15, 1.60 ± 0.15, 1.73 ± 0.18) compared to the fruit peel of crack-susceptible material “812” (0.15 ± 0.01, 0.38 ± 0.04, 0.42 ± 0.04, 0.59 ± 0.05, 1.00 ± 0.11), respectively. However, the lignin content in watermelon material “1061” seemed lower at 14 DAP due to the cell structure differences, but steadily increased from 14–28 DAP; however, the lignin content became stable until 35 DAP. The lignin content in watermelon material “812” gradually increased at each period.

### 2.11. Expression Patterns of ClPRXs in Different Tissues

**Figure 11:**
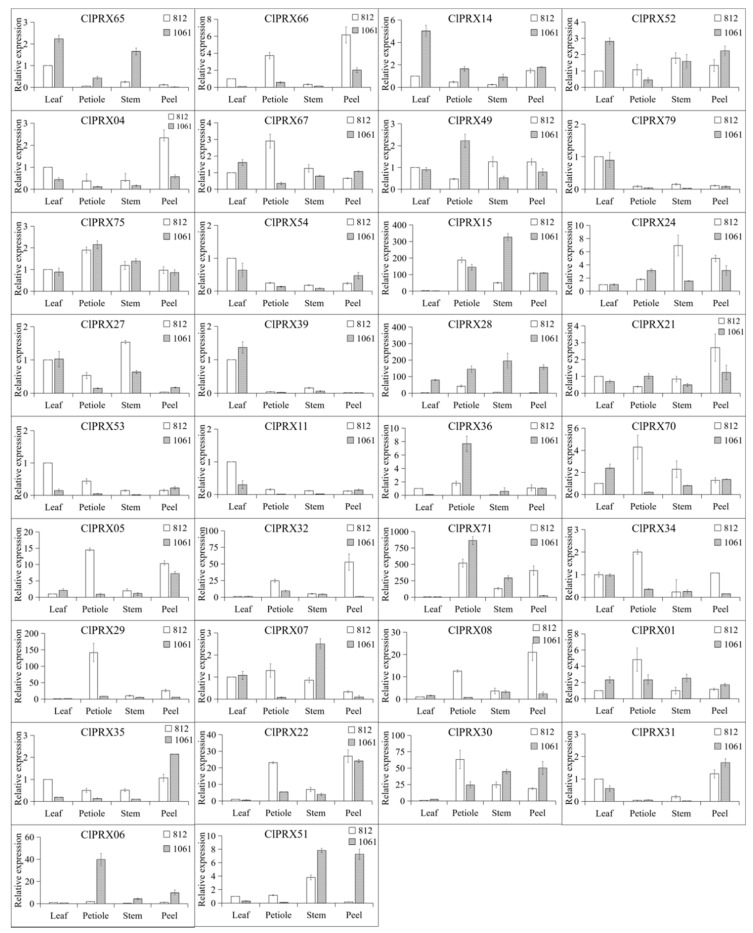
Expression levels of *ClPRXs* in different tissues. The values represent the means ± SDs, and different bars indicate significant differences (*p* < 0.05) of genes’ expression level.

To further explore the peel lignin accumulation difference, we determined the expression patterns of ClPRX genes between two different watermelon materials. Higher expression was observed in group A and lower expression in group B (Figure 9). A total of 34 ClPRX expression patterns were analyzed in different tissues (leaf, petiole, stem, and peel) by qRT-PCR (Figure 11). The obtained results indicated that 8 ClPRXs (*ClPRX65*, *ClPRX14*, *ClPRX52*, *ClPRX79*, *ClPRX54*, *ClPRX39*, *ClPRX53*, *ClPRX11*) were highly expressed in leaves, 12 ClPRXs were highly expressed in petioles (*ClPRX67*, *ClPRX49*, *ClPRX75*, *ClPRX36*, *ClPRX70*, *ClPRX71*, *ClPRX34*, *ClPRX29*, *ClPRX06*, *ClPRX01*, *ClPRX05*, *ClPRX30*), 6 ClPRXs were highly expressed in stems (*ClPRX15*, *ClPRX24*, *ClPRX27*, *ClPRX28*, *ClPRX07*, *ClPRX51*), and 8 ClPRXs were highly expressed in peel tissue (*ClPRX66, ClPRX04*, *ClPRX21*, *ClPRX22*, *ClPRX32*, *ClPRX08*, *ClPRX35*, *ClPRX31*). These ClPRX genes were strongly expressed in four different tissues, which might play a molecular role in the development and function of different watermelon tissues.

Six *ClPRXs* (*ClPRX54*, *ClPRX28*, *ClPRX35*, *ClPRX30*, *ClPRX06*, and *ClPRX51*) had significantly higher gene expression in “1061” than in “812”. Among them, *ClPRX06* and *ClPRX51* were located in the same phylogenetic tree branch as *AT5G66390.1* (*AtPRX72*) in Arabidopsis (Figure 5), and colinear gene pairs existed (Table S4), further supporting their similar functions. Previous studies have shown that *AT5G66390.1* (*AtPRX72*) plays an important role in lignin biosynthesis [16,23]. *ClPRX54* is an independent branch of group VII watermelon in the phylogenetic tree, suggesting that *ClPRX54* might play a unique role in watermelon crops. These three genes are considered candidate genes for lignin synthesis in the peel of the materials, and further studies of the gene expression during different periods of peel development are needed.

### 2.12. Expression Patterns of Candidate ClPRXs at Different Developmental Stages of Peel

**Figure 12:**
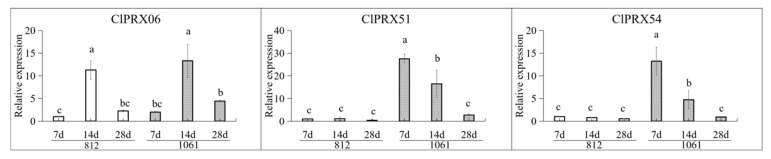
Expression levels of candidate *ClPRXs* in different developmental stages of peel. The values represent the means ± SDs, and different bars indicate significant differences (*p* < 0.05) of genes’ expression level. Statistical letters (a, b, c, and bc) indicate the significant differences of relative expression of genes in “812” and “1061” watermelon materials.

To further study the difference in ClPRX expression levels in different stages of watermelon peel development, we performed qRT-PCR analysis to examine the expression of three candidate genes (Figure 12). Among the three candidate ClPRXs, both the *ClPRX51* and *ClPRX54* genes were significantly under expressed in the material with less lignin accumulation “812”, while they were strongly expressed in material “1061” with more lignin accumulation that could form stone cell tissue, showing a declining trend in different periods. There was a significant difference in lignin accumulation, especially in the early stage (7 DAP, 14 DAP). However, *ClPRX06* showed a trend of first increasing and then decreasing in the three periods of the two different materials. The expression level of material “1061” was higher than that of material “812” in the same period, but the difference between varieties in the same period did not reach significance. Therefore, the *ClPRX51* and *ClPRX54* genes are most likely to be involved in the synthesis of lignin in watermelon peel.

## 3. Discussion

Among the PRXs, class III peroxidases are well known on a large scale in plants; they are usually secreted into the cell wall or into the cytoplasmic base fluid and vacuoles and are widely involved in the physiological process of plant growth and development [23,24]. The PRX members of Arabidopsis, rice, maize, pear, cassava, potato, birch, and other plants have been identified and analyzed, but a detailed study of the watermelon PRX gene family has not been reported to date. Analyses of the genome-wide characteristics of ClPRX based on genome-wide watermelon database information could provide information for molecular improvement of watermelon quality in the future.

In this study, a total of 79 ClPRXs were identified based on the genomic information of *Citrullus lanatus Subsp. vulgaris CV. 97103*. The number of ClPRXs was mostly similar to that of Arabidopsis (73) [5], but lower than that of rice (138) [6] and maize (119) [7], a difference that can be explained by the status of Watermelon and Arabidopsis as dicotyledons and the close relationship of PRX family members to each other compared with rice and maize. The present results are also consistent with the finding that the PRX gene family extension differs between monocotyledons and eudicotyledons [7] A total of 47 collinear gene pairs were found between 35 PRX genes in Arabidopsis and 28 PRX genes in watermelon, among which the Arabidopsis PRX gene could map 1–4 watermelon PRX genes. Furthermore, a study on the gene replication relationship of ClPRXs reported 12 pairs of fragment replication genes and a total of 25 tandem duplicated genes. According to the above analysis, some ClPRXs were derived from the purification and selection of the PRX gene in the evolutionary process, and the gene sequence was highly conserved, maintaining the functional stability of the ClPRX protein. The other part exhibited fragment replication and tandem replication during the evolution of the watermelon genome. According to the Arabidopsis PRX gene family, there were at least four replicates, which may be the key reason for the amplification and functional differentiation of ClPRXs [6,25]. Our results provide significant new resources for understanding the evolution of the PRX gene family in different species.

It is well known that multigene family evolution leads to the diversification of gene structures, including protein sequences, exons and introns, promoters, and enhancers [26]. In this study, the structure of the ClPRX gene was studied, and it was found that these genes had a large number and different lengths of gene sequences. The comparison of primary structural sequences revealed a high variability in watermelon materials. Previous studies have also found a homology of total amino acid sequences of PRX gene family members in multiple specimens of less than 35% [5,27], but due to its coding region and motif research, we found that high similarity, indicating a relatively conserved status, was present in a number of structural domains (distal heme combined with domain structure, location structure, and proximal heme combining structural domains), including the heme binding site distal histidine (Hd) and proximal histidine (HP). Eight cysteines (C1–C8) are key amino acids in the disulfide bonds forming the secondary structure of peroxidase [28,29]. These PRX proteins have similar sequence structures only in specific domains, but not in other genetic regions, so we hypothesized that nonhomologous segments define their functions.

In addition, the ClPRX gene family was divided into seven groups based on the phylogenetic tree analysis. The gene structure (exon/intron) and motif composition of the same group were relatively conserved, indicating that genes in the same group might be more similar in function. Then, the ClPRXs and Arabidopsis PRX gene family members were analyzed using the constructed phylogenetic tree so that the ClPRX gene family could be divided into seven groups and eleven subclasses. Based on the existing functions of Arabidopsis on the same branch, we could better predict the function of ClPRXs. The ClPRXs contained a unique group VII (also subclass A) in watermelon, and group VII had the most distant evolutionary relationship. In accordance with the results shown in Figure 3A, compared with the other six groups, the gene structure of this group contained a peroxidase conserved domain, but a large number of introns were present in the gene structure. Moreover, the protein conserved motif was mostly missing. Studies have shown that introns were specifically inserted into the plant genome during plant evolution and were retained [30], which enabled these genes to undergo faster differentiation and pseudogenification [31].

Therefore, PRX genes of subclass A, as genes unique to watermelon, may have more important functions, which requires further study. In addition, members of the watermelon PRX gene promoter region family were found, as well as various gene family members mainly involved in temperature sensing (photosensitive, low temperature, drought), hormone regulation (auxin, gibberellin, abscisic acid, salicylic acid), growth and development (zein metabolic regulation, the biosynthesis of flavonoids), and plant defense and stress reactions, among other activities [32]. Studies have shown that protein sequences with similar structures and functions may exhibit different expression levels due to differences in regulatory sequences, which may be the result of long-term adaptation to environmental changes in plants [12]. Therefore, studying promoter homeopathic elements is one of the keys to comprehensively understanding gene-specific expression.

The accumulation of lignin in the plant cell wall results in thickening of the cell wall and wood (stem) hardening. This lignification is one of the important functions of the third type of peroxidase. In this study, we found that members of the ClPRX gene family were widely involved in the phenylpropanoid biosynthesis pathway in KEGG, accounting for 68/75 genes. Previous studies have shown that peroxidase (EC1.11.1.7) is the last reactive enzyme in the synthesis of large molecules of lignin via the polymerization of small molecules (coumaryl alcohol, coniferyl alcohol, and sinapyl alcohol) of lignin in the phenylpropanoid biosynthesis pathway, and the lignification process of plants occurs throughout the growth period of plants [14,15]. *AtPrx2*, *AtPrx25*, *AtPrx71*, and *AtPrx72* play an important role in Arabidopsis lignification [16,17]. In our previous research, we found significant differences in the stone cell structure caused by lignification between the two materials with obvious differences in peel hardness, and no stone cell structure was detected in the materials with low peel hardness, which were susceptible to cracking [22]. In the latest study, herbaceous peony increased the layers of thickened secondary cells and lignin accumulation, resulting in enhanced stem strength and demonstrably straight stems [33]. There was a positive correlation between lignin, cellulose, and stone cell contents in 206 sand pear cultivars [34]. Therefore, we speculated that low lignin synthesis might lead to low peel hardness and easy cracking.

In this study, watermelon peel hardness was significantly correlated with stone cell development and lignin accumulation, and a significant correlation was identified between peel hardness and stone cell structure development at 14 DAP, 21 DAP, and 28 DAP. There was also a significant difference in endogenous lignin content between the two materials in each stage. Basically, stone cell development is the process of lignification caused by lignin accumulation in the cell wall, which can effectively enhance the hardness of tissue. We also obtained two watermelon peel materials with significant differences in endogenous lignin accumulation (28 DAP), which were subjected to qRT-PCR expression analysis. We screened six ClPRX genes (*ClPRX54*, *ClPRX28*, *ClPRX35*, *ClPRX30*, *ClPRX06*, *ClPRX51*). The expression of these genes was significantly higher in the “1061” peel material (with high lignin content) than the “812” peel material (with low lignin content). *ClPRX51* and *ClPRX54* showed significantly lower expression in the material with less lignin accumulation of “812” material, but strong expression in the material with more lignin accumulation capable of forming stone cell tissue of “1061” material, and they showed a declining trend in different periods. The significant differences were particularly observed in the early stage of lignin accumulation (7 DAP, 14 DAP), which may have represented the key genes affecting lignin synthesis in watermelon peel.

In particular, the *ClPRX51* gene and Arabidopsis *AT5G66390.1* gene were on the same phylogenetic tree branch (see Figure 5), and colinear gene pairs (Table S2) were identified in both of them, suggesting their similar functions. In previously published studies, knockout of the Arabidopsis gene (*AT5G66390.1*) exhibited significant reduction of lignin contents compared with the wild-type (WT) [23]. Our study also revealed that the *ClPRX51* gene has similar functional effects regulating the lignin synthesis-related pathways in watermelon peel and needs to be further studied.

## 4. Materials and Methods

### 4.1. Plant Materials and Sampling

Two different types of watermelon parent materials, “1061” and “812”, with distinct peel characteristics were planted in a plastic greenhouse at XiangYang Experimental Base of Northeast Agricultural University, Harbin, China. The differential peel characteristics of both materials have been described in our previously published study [21].

The plants of two watermelon materials were grown and checked daily for finding the new sprouting buds. When plants reached the full flowering stage, then selected flowers (to be opened the next morning) were marked and covered with a paper cap. The manual pollination was performed in the next morning (6.00 a.m. to 10.00 a.m.), and each pollinated flower was protected with the same paper cap and labeled with the date, to observe the developmental stages and fruit maturity at days after pollination (DAP). Peel materials were sampled at 7 DAP, 14 DAP, 21 DAP, 28 DAP, and 35 DAP, and subsequently, the peel hardness, peel cell microstructure, and peel endogenous lignin contents were observed, to study the lignin accumulation at different developmental stages. For the qRT-PCR analysis of ClPRXs, fruit peels were collected for each key time period of different developmental stages at 7 DAP, 14 DAP, and 28 DAP (when stone cell formation started). In addition, peel materials of 28 DAP and nearby stem, petiole, and leaf materials were obtained for RNA extraction, cDNA synthesis, and qRT-PCR analysis of ClPRXs.

In brief, all the experimental materials from the vegetative and reproductive growth stages of each plant were collected with 3 replications. A total of 1 g leaf, petiole, stem, and peel tissue material was sampled from the marked position of the second female flower. A total 3 undamaged and random samples from each fruit were also collected at 5 different stages with each time interval of 7 DAP until 35 DAP. For fruit peel hardness analysis, three random peel samples (3 cm × 3 cm × 3 cm) were also collected from each fruit, and texture analysis (TA-XT Plus) (Stable Micro System Division, U.K.) was performed. The peel was separated from the fruit flesh and cut into equal samples of 5 mm × 5 mm × 3 mm. Paraffin sectioning was performed by embedding the peel samples into FAA (1:1:18 mixture (*v/v/v*) of formalin:acetic acid:50% ethanol) fixative solution, and the endogenous lignin contents were visually quantified. In addition, total RNA extraction was also performed from subsequent frozen samples using the TRIzol method [35].

### 4.2. Preliminary Search and Identification of ClPRXs in the Watermelon Genome

Watermelon peroxidase gene family members (ClPRXs) were initially searched for identification, and three methods (searching keywords, HMM, Blast) were used to obtain the gene information and the preliminary screening. The ClPRX genes were termed according to the naming method of Arabidopsis as reported previously [5], e.g., the letter “Cl” for *Citrullus lanatus* (Thunb.) Matsum. et Nakai and “PRX” for peroxidase and by a number indicating the position of genes on the respective chromosomes.

The preliminary data of ClPRX members was retrieved on April 5, 2021, as follows: (1) search the keyword “peroxidase” in the Cucurbitaceae database (http://cucurbitgenomics.org/ (accessed on 2 April 2021)); (2) download the peroxidase protein conserved domain model (PF00141) from the Pfam library (http://pfam.xfam.org/ (accessed on 2 April 2021)) using HMMER 3.0_Windows software (http://hmmer.org/ (accessed on 2 April 2021)) [36], followed by the application of Pfam (PF00141) as a template to search and compare the reference watermelon V2 proteome sequence database (e-value ≤ 10^−5^); (3) accession over Arabidopsis exhibiting 73 peroxidase gene protein sequences [37] in the Cucurbitaceae database (http://cucurbitgenomics.org/ (accessed on 2 April 2021)), followed by application of the BlASTP method. The preliminary screening steps involved three subsequent steps: (1) removal and merging of duplicated genes to preserve genetic information; (2) analysis and visualization of protein motifs to select the genes with obvious differences in protein motifs and further detection using TBtools software [38] and the online MEME (http://meme-suite.org/tools/meme (accessed on 2 April 2021)); (3) retrieval of protein domain conservative projections through the online NCBI-CD (https://www.ncbi.nlm.nih.gov/Structure/cdd/wrpsb.cgi (accessed on 2 April 2021)) database and removal of functional domains of incomplete proteins to eventually determine the candidate ClPRX members.

ClPRX genetic information (the length of the chromosome location, encoding, CDS, exons) in the Cucurbitaceae database (http://cucurbitgenomics.org/ (accessed on 2 April 2021)) was performed using the online software ExPASy ProtParam tools (http://web.expasy.org/protparam/ (accessed on 2 April 2021)) [38], and basic information was obtained to predict the protein (amino acid (aa) length, molecular weight (MW), and isoelectric point (PI)). Online information at http://h-s.p-443.npsa-prabi.ibcp.fr.neau.vpn358.com/cgi-bin/npsa_automat.pl?page=/NPSA/npsa_gor4.html (accessed on 2 April 2021) for protein secondary structure prediction was retrieved using softberry web online tools (http://linux1.softberry.com/berryPHTML?Topic=protcomppl&group=programs&group=Proloc (accessed on 2 April 2021)) and finally used to predict the subcellular localization of 79 ClPRX proteins.

### 4.3. Chromosome Localization, Gene Replication Relationship, and Interspecific and Intraspecific Collinearity Analysis

The One Step MCScanX module in the TBtools software [26] was used to obtain genome-wide replication events and identify the gene replication relationship of ClPRXs. Genome-wide collinearity analysis was performed among watermelon and watermelon intraspecies, watermelon, and Arabidopsis. The “Amazing Super Circos” module was used to visualize the gene localization and the gene linear relationship on the respective chromosomes. The “Ka/Ks Calculator” module in the TBtools software was used to further calculate the evolutionary relationship between peroxidase genes and fragment replication and tandem replication by incorporating the Ka and Ks of watermelon intraspecific peroxidase genes and comparative interspecific peroxidase genes of watermelon and Arabidopsis. The pressure selection analysis was carried out by calculating the Ka/Ks ratio.

### 4.4. ClPRXs’ Structure and Protein Domain Analysis

Complete gene structure information for PRX, including gene length, CDS position, and gene functional domain prediction information, was obtained using the “Gene Structure View” module of the TBtools software. Then, the MEME online database (http://meme-suite.org/ (accessed on 2 April 2021)) was used to analyze the identified watermelon PRX protein conserved motifs. The motif length ranged from 6-200 amino acids; the number of motifs was set to 20; the analysis results were saved. Multisequence alignments of the PRX protein were filtered with MEGA 7.0 software in Muscle [39], and the comparison results were imported into the TBtools module “Quick Run TrimAL”. Then, the composition of the parameter selection “NJ_STRICTPLUS” was used to display the results of the amino acid sequence alignment. The NCBI-CD (https://www.ncbi.nlm.nih.gov/Structure/cdd/WRPSB.Cgi (accessed on 2 April 2021)) functional domain and domain site analysis, functional domain binding site information, and NCBI functional module “Cn3D macromolecular structure viewer” were used to demonstrate the functional domain binding sites.

### 4.5. Construction of the Phylogenetic Tree for Watermelon and Arabidopsis

The obtained protein sequences of the watermelon and Arabidopsis PRX families along with homologous protein sequences were arranged in the MEGA 7.0.26 software, and an evolutionary tree was constructed. The neighbor-joining (NJ) adjacent method and 1000 bootstraps were used, and the other parameters were also set as default values for final construction of the evolutionary tree.

### 4.6. Functional Enrichment Analysis of ClPRXs

Gene functional enrichment analysis was performed using the online database (http://cucurbitgenomics.org/goenrich (accessed on 2 April 2021)) for ClPRX GO annotations using the online TBtools software [26] “GO Enrichment” module downloaded GO annotation-based package hierarchy (GO-basic. Obo) and the GO Enrichment function. KEGG enrichment analysis was performed using the online KEGG database (http://cucurbitgenomics.org/pwyenrich (accessed on 2 April 2021)) for ClPRX annotation and the downloaded KEGG Enrichment using the “KEGG Enrichment” module. Finally, the “Enrichment Bar Plot” module was used to visually display the results.

### 4.7. Transcriptome Analysis of ClPRXs at Different Periods of Peel and Flesh

The online watermelon database (http://cucurbitgenomics.org/ (accessed on 2 April 2021)) and transcriptome registration number SRP012849 [40] were searched, and the downloaded ClPRXs of gene expression data were used to construct the heatmap using the TBtools software.

### 4.8. RNA Extraction, cDNA Synthesis, and Gene Expression Analysis

The samples were lyophilized at 72 h using a lyophilizer, and RNA was extracted using the common TRIzol method [35]. Total RNA was imaged on a 1% agarose gel, and the RNA concentration was detected using a NanoPhotometer^®^ P330 (IMPLEN, Munich, Germany). Then, the PrimeScript RT Master Mix Perfect Real-Time kit (TOYOBO, Osaka, Japan) was used to synthesize first-strand cDNA with 1 μg RNA. The final cDNA was placed in a –20 °C refrigerator for quantitative RT-PCR (qRT-PCR).

The *Cla020175* gene was used as the reference gene, and all the gene primers used in the qRT-PCR analysis are shown in Table S2. The Primer Premier (V6.0) software was used to design the gene-specific primers [41]. Three biological replicates and three technical replicates were used for each cultivar tissue assayed. A 20 μL PCR mixture was prepared with 10 μL of SYBR Green Master mix (TOYOBO, Osaka, Japan), 1 μL of each primer pair, and 1 μL of cDNA templates. PCR amplification of target genes was performed in 96-well optical reaction plates on an iQ5 Gradient Real Time PCR system (Analytik, Jena, Germany). The PCR assay was set as follows: 95 °C for 60 s; 40 cycles of 95 °C for 15 s, 58 °C for 20 s, and 72 °C for 15 s; a final melt curve analysis in which the temperature was increased from 55 °C to 95 °C at a rate of 0.5 °C/5 s; a final hold at 4 °C. The specificity was verified by melt curve analysis and agarose gel electrophoresis of the qRT-PCR products. The relative expression levels of all different genes were determined using the 2^−ΔΔCT^ method [42].

### 4.9. Determination of Watermelon Peel Hardness and Paraffin Sectioning

For the peel hardness, a 2 mm (P/2) diameter probe TA-XT Plus texture instrument was used to conduct puncture tests on the ripened fruit peel of different watermelon varieties as reported previously [21]. The paraffin-embedded tissue section sampling and preparation method were used as previously reported [31]. In brief, the material was embedded into FAA (1:1:18) fixed solution, which was changed for overnight incubation and dehydrated in an alcohol concentration gradient. The xylene was transparent after addition to the paraffin clastic and paraffin embedding at 65 °C using turning-wheel microtome sectioning at a slice thickness of 8 μm, drying under xylene concentration gradient dewaxing, an alcohol concentration gradient, red and green dye fixation, sealing, and imaging.

### 4.10. Endogenous Quantification of Lignin Contents in Watermelon Peel

A total of 1 g of leaf material was ground in liquid nitrogen; 0.1 g was weighed and put into a 2 mL precooled centrifugal tube, and then, the amount of lignin in the watermelon peel was determined by the thioglycolic acid method [43]. Simultaneously, commercial lignin (alkaline spruce lignin, Aldrich, Milwaukee, WI, USA) was used to configure standard samples with different gradient concentrations. The final concentration of all standard samples was determined using the same method, which was used to calibrate the lignin content curve and to calculate the endogenous lignin contents in both watermelon peel materials.

## 5. Conclusions

In this study, a total of 79 ClPRX members were identified by genome-wide bioinformatics analysis of watermelon. These identified genes were divided into seven groups and eleven subclasses based on the constructed phylogenetic tree. To better understand these genes, we also analyzed the chromosome distribution, gene replication, gene structure, conserved motifs, GO annotation, KEGG pathway, and promoter element prediction and combined them with the expression analysis of the ClPRX gene family in fruits using online transcriptome data. In addition, we focused on the key role of the peel lignin synthesis-related ClPRX gene family, and qRT-PCR was used to analyze the expression patterns in different tissues during different development periods. The obtained results showed that deletion and replication events occurred in the process of gene amplification of the ClPRX family, and the overall protein sequences had low homology, but consistently, four conserved functional loci were present. ClPRXs showed different tissue-specific abiotic stress and hormone response expression patterns, providing strong evidence that the ClPRX gene family participates in a variety of different physiological processes occurring in plants. To our knowledge, this is the first study to predict the key roles of two ClPRXs in peel lignin synthesis. In conclusion, the extensive data collected in this study can be used for additional functional analyses of ClPRXs in watermelon growth and development and hormone and abiotic stress responses.

## Figures and Tables

**Table 1:** The detailed genes information of ClPRXs.

Gene Symbol	Gene ID	Chromosome	Starting Point	End Position	Length of Coding Region	Length of CDS	Number of Exons	Coding Protein Characteristics					
								Amino Acid (aa) Length	Molecular Weight (MW)/ 1000-Dalton (kDa)	Isoelectric Point (PI)	Secondary Structure Characteristics		
											α-Helix (%)	β-Sheet (%)	Random Coil (%)
*ClPRX01*	Cla97C01G005010.1	1	4723552	4726977	3426	1011	4	336	35,957.3	4.42	25.30	14.58	60.12
*ClPRX02*	Cla97C01G011510.1	1	20830864	20835096	4233	975	4	324	35,565.6	5.58	29.32	14.51	56.17
*ClPRX03*	Cla97C01G013060.1	1	26885053	26886313	1261	1005	2	334	37,268.8	6.17	23.95	19.16	56.89
*ClPRX04*	Cla97C01G015760.1	1	29495599	29497136	1538	984	4	327	36,838.1	6.88	33.03	17.43	49.54
*ClPRX05*	Cla97C01G016570.1	1	30277513	30278818	1306	987	4	328	35,737.8	9.14	30.79	19.21	50.00
*ClPRX06*	Cla97C01G019360.1	1	32325769	32328138	2370	1002	4	333	36,365.2	9.05	27.33	16.82	55.86
*ClPRX07*	Cla97C01G019890.1	1	32769843	32773816	3974	891	9	296	32,914.6	8.30	38.18	12.50	49.32
*ClPRX08*	Cla97C01G020000.1	1	32847704	32849046	1343	960	4	319	34,676.6	9.14	33.86	8.78	57.37
*ClPRX09*	Cla97C02G028190.1	2	1675720	1677245	1526	978	4	325	35,449.7	8.62	31.38	17.85	50.77
*ClPRX10*	Cla97C02G028200.1	2	1681804	1692106	10303	1755	9	584	64,150.5	8.95	26.54	20.38	53.08
*ClPRX11*	Cla97C02G030990.1	2	3941579	3945614	4036	861	9	286	31,559.9	6.67	40.21	10.14	49.65
*ClPRX12*	Cla97C02G035070.1	2	10386004	10387325	1322	987	4	328	35,847.9	8.09	39.02	13.41	47.56
*ClPRX13*	Cla97C02G037820.1	2	24964862	24967129	2268	1005	4	334	36,089.3	8.86	27.84	20.66	51.50
*ClPRX14*	Cla97C02G044750.1	2	32912405	32914360	1956	1065	2	354	39,631.8	9.21	32.49	12.71	54.80
*ClPRX15*	Cla97C02G045070.1	2	33199676	33201140	1465	981	4	326	35,326.4	9.38	22.39	19.02	58.59
*ClPRX16*	Cla97C02G045080.1	2	33206687	33208047	1361	870	4	289	31,399.8	8.72	24.22	19.38	56.40
*ClPRX17*	Cla97C02G045090.1	2	33211116	33212531	1416	975	4	324	35,542.8	9.41	32.41	15.43	52.16
*ClPRX18*	Cla97C02G045100.1	2	33215577	33216865	1289	942	4	313	34,070.4	5.47	23.96	17.25	58.79
*ClPRX19*	Cla97C02G045120.1	2	33230817	33232144	1328	960	3	319	34,792.8	9.27	24.76	21.63	53.61
*ClPRX20*	Cla97C02G045130.1	2	33237622	33238928	1307	960	3	319	34,574.3	8.53	21.94	19.75	58.31
*ClPRX21*	Cla97C02G046770.1	2	34511814	34515485	3672	753	9	250	27,274.9	5.60	43.60	8.40	48.00
*ClPRX22*	Cla97C02G049870.1	2	37212086	37215158	3073	1020	4	339	36,812.2	7.53	26.84	21.83	51.33
*ClPRX23*	Cla97C02G049880.1	2	37219380	37220760	1381	1014	4	337	36,152.5	4.73	22.26	18.99	58.75
*ClPRX24*	Cla97C02G049890.1	2	37226317	37227586	1270	1023	4	340	36,696.6	8.60	26.47	16.18	57.35
*ClPRX25*	Cla97C02G049900.1	2	37234294	37235546	1253	993	3	330	35,335.4	5.71	33.64	15.45	50.91
*ClPRX26*	Cla97C02G049910.1	2	37238447	37239716	1270	990	3	329	35,869.1	9.07	32.83	12.46	54.71
*ClPRX27*	Cla97C02G049920.1	2	37245856	37260161	14306	990	4	329	36,370.9	5.30	27.96	14.29	57.75
*ClPRX28*	Cla97C02G049930.1	2	37271693	37272975	1283	1026	4	341	36,810.4	6.53	31.38	13.78	54.84
*ClPRX29*	Cla97C02G049940.1	2	37280732	37282786	2055	960	4	319	34,277.4	4.60	24.76	18.81	56.43
*ClPRX30*	Cla97C02G049950.1	2	37290261	37291772	1512	963	4	320	34,345.3	4.70	23.12	23.75	53.12
*ClPRX31*	Cla97C03G051030.1	3	255043	256982	1940	945	4	314	33,950.3	5.75	29.94	16.24	53.82
*ClPRX32*	Cla97C03G053010.1	3	2050061	2058477	8417	1899	14	632	71,578.4	5.41	40.19	12.97	46.84
*ClPRX33*	Cla97C03G055260.1	3	4235106	4238033	2928	1029	3	342	37,922.2	5.75	29.82	16.08	54.09
*ClPRX34*	Cla97C03G055890.1	3	4781189	4782652	1464	996	4	331	37,689.3	8.66	40.48	11.78	47.73
*ClPRX35*	Cla97C03G059200.1	3	8602556	8605339	2784	1044	3	347	37,903.6	9.27	37.75	14.41	47.84
*ClPRX36*	Cla97C04G070210.1	4	10427630	10432305	4676	960	4	319	35,032.0	8.10	31.35	18.18	50.47
*ClPRX37*	Cla97C04G075580.1	4	23076560	23078327	1768	894	4	297	33,467.3	5.70	37.71	12.46	49.83
*ClPRX38*	Cla97C05G089640.1	5	7906134	7907486	1353	918	3	305	33,412.1	8.04	32.79	14.10	53.11
*ClPRX39*	Cla97C05G097050.1	5	26403410	26407012	3603	986	3	328	34,981.4	5.21	17.90	27.10	54.80
*ClPRX40*	Cla97C05G098800.1	5	27994171	27995541	1371	963	4	320	35,772.8	9.05	26.80	18.10	55.00
*ClPRX41*	Cla97C05G105280.1	5	32937084	32940587	3504	1020	4	339	37,654.1	5.28	32.10	15.60	52.20
*ClPRX42*	Cla97C05G107830.1	5	34625160	34627681	2522	1032	2	343	37,943.1	5.24	32.60	16.90	50.40
*ClPRX43*	Cla97C06G110270.1	6	920898	925297	4400	1017	4	338	37,923.6	9.08	35.80	14.20	50.00
*ClPRX44*	Cla97C06G113900.1	6	4822850	4824110	1261	1014	4	337	36,341.1	4.93	33.20	12.70	54.00
*ClPRX45*	Cla97C06G115010.1	6	5937272	5940105	2834	995	4	329	36,275.5	9.47	34.30	13.30	52.20
*ClPRX46*	Cla97C06G115020.1	6	5956825	5959012	2188	990	4	329	36,069.2	8.37	28.80	16.40	54.70
*ClPRX47*	Cla97C06G120340.1	6	22349470	22352615	3146	978	4	325	35,998.0	6.95	33.50	11.00	55.30
*ClPRX48*	Cla97C06G120350.1	6	22368798	22371140	2343	963	4	320	35,447.3	5.62	28.70	18.10	53.10
*ClPRX49*	Cla97C06G126320.1	6	28110200	28111195	996	996	1	331	36,316.6	6.95	37.70	12.60	49.50
*ClPRX50*	Cla97C07G131030.1	7	2626605	2628423	1819	799	3	315	34,150.5	8.79	29.50	14.60	55.80
*ClPRX51*	Cla97C07G135790.1	7	21625198	21627270	2073	1004	4	333	36,548.5	9.13	36.60	12.30	51.00
*ClPRX52*	Cla97C07G144420.1	7	31806093	31807972	1880	1024	3	339	38,007.4	6.01	25.60	16.80	57.50
*ClPRX53*	Cla97C08G148570.1	8	16719601	16725763	6163	1710	12	457	49,685.2	7.63	31.90	12.20	55.80
*ClPRX54*	Cla97C08G156740.1	8	24456600	24460113	3514	1011	10	336	36,670.9	6.97	33.60	13.90	52.30
*ClPRX55*	Cla97C08G157670.1	8	25164283	25165558	1276	954	4	317	34,657.7	9.31	34.70	11.00	54.20
*ClPRX56*	Cla97C09G167060.1	9	3951866	3953193	1328	999	2	331	36,014.3	9.57	36.50	15.70	47.70
*ClPRX57*	Cla97C09G167070.1	9	3957503	3958980	1478	1008	2	335	36,338.0	6.39	34.90	15.20	49.80
*ClPRX58*	Cla97C09G167080.1	9	3961741	3963120	1380	1020	3	339	36,849.0	9.00	27.10	21.50	51.30
*ClPRX59*	Cla97C09G167090.1	9	3968829	3971128	2300	1011	3	336	36,458.7	9.21	31.80	18.70	49.40
*ClPRX60*	Cla97C09G167100.1	9	3973203	3974880	1678	1008	2	335	36,590.7	5.84	27.40	16.40	56.10
*ClPRX61*	Cla97C09G167110.1	9	3978060	3980910	2851	987	2	328	36,322.7	8.37	34.70	11.80	53.30
*ClPRX62*	Cla97C09G167120.1	9	3983474	3985318	1845	999	2	332	35,972.0	8.36	27.10	18.00	54.80
*ClPRX63*	Cla97C09G175150.1	9	13236273	13238337	2065	998	4	331	37,419.7	5.94	23.20	23.80	52.80
*ClPRX64*	Cla97C09G177060.1	9	26136385	26142213	5829	990	4	329	35,419.4	5.64	33.70	13.30	52.80
*ClPRX65*	Cla97C09G177290.1	9	27493392	27495394	2003	989	3	329	35,713.6	8.39	19.40	24.00	56.50
*ClPRX66*	Cla97C09G177300.1	9	27526270	27528397	2128	987	4	328	36,178.3	8.11	21.00	24.70	54.20
*ClPRX67*	Cla97C09G178920.1	9	32289531	32291626	2096	1044	4	347	38,431.5	4.93	30.20	14.40	55.30
*ClPRX68*	Cla97C09G180150.1	9	33769566	33770531	966	966	1	321	35,223.1	8.06	32.70	13.40	53.80
*ClPRX69*	Cla97C09G184240.1	9	37364363	37365922	1560	1170	4	389	43,464.0	5.18	32.10	14.90	52.90
*ClPRX70*	Cla97C10G197400.1	10	27216069	27217049	981	983	1	326	35,890.8	8.33	28.20	12.80	58.90
*ClPRX71*	Cla97C10G203590.1	10	33215489	33219675	4187	969	5	322	36,313.8	7.58	30.10	13.30	56.50
*ClPRX72*	Cla97C11G206940.1	11	765876	767014	1139	951	3	316	34,727.6	9.36	25.90	19.30	54.70
*ClPRX73*	Cla97C11G207220.1	11	1081415	1082545	1131	945	3	314	33,974.5	8.07	24.80	20.00	55.10
*ClPRX74*	Cla97C11G207230.1	11	1086466	1087611	1146	954	3	317	34,413.2	8.36	26.80	20.80	52.30
*ClPRX75*	Cla97C11G212730.1	11	6071711	6081296	9586	7195	9	564	61,162.5	9.10	25.80	10.40	63.60
*ClPRX76*	Cla97C11G214520.1	11	7856001	7857609	1609	972	3	323	34,727.5	5.35	35.60	16.40	47.90
*ClPRX77*	Cla97C11G214530.1	11	7867036	7870803	3768	927	2	308	33,039.3	6.90	25.00	19.40	55.50
*ClPRX78*	Cla97C11G214540.1	11	7899468	7901107	1640	984	3	327	35,316.9	4.90	34.80	15.20	49.80
*ClPRX79*	Cla97C11G220240.1	11	26276590	26282064	5475	1050	11	349	38,057.1	6.06	36.30	8.60	55.00

## Data Availability

Available upon request from the corresponding author.

## Supplementary Materials

The following are available online at https://www.mdpi.com/article/10.3390/ijms23020642/s1.
